# 4061 Clinical utility of precision medicine approaches to guide anti-platelet selection for adult patients with acute coronary syndromes (ACS), following percutaneous coronary intervention (PCI)

**DOI:** 10.1017/cts.2020.328

**Published:** 2020-07-29

**Authors:** Scott Mosley, Samantha Yeung, David Shavelle, Leonardo Clavijo, Tien Ng

**Affiliations:** 1 University of Southern California School of Pharmacy; 2 Keck School of Medicine at University of Southern California

## Abstract

OBJECTIVES/GOALS: Our goal is to determine if a precision medicine approach to guide anti-platelet therapy for patients with ACS, post PCI, is feasible for a diverse urban population. Also, we will evaluate if guided therapy reduces major adverse cardiovascular events (MACE) while remaining economically sustainable. METHODS/STUDY POPULATION: This prospective, pragmatic study will enroll two-hundred patients with ACS undergoing PCI, receiving DAPT. Patients will receive point-of-care *CYP2C19* genotyping. Patients with at least one loss-of-function (LoF) allele will be recommended prasugrel. Those without LoF alleles, will be recommended to take prasugrel for 7 days then clopidogrel for 7 days, followed with platelet reactivity phenotyping. Patients with HPR >208 P2Y_12_ reaction units will take prasugrel; the remainder will take clopidogrel. We will review electronic health records and contact patients at baseline, then at 1, 3, 6, and 12 months to collect data for cardiovascular and health-related quality of life (HRQoL) outcomes. RESULTS/ANTICIPATED RESULTS: Feasibility and clinical utility will be measured by the proportion of patients with a genotype or phenotype leading to a clinical recommendation of alternative therapy and whether or not recommendations were accepted by clinicians. Effectiveness will be measured by combined MACE (composite of cardiovascular death, non-fatal myocardial infarction, and non-fatal stroke), stent thrombosis, and major and minor bleeding over the study period. Cost of testing, 30-day hospital readmission, and HRQoL questionnaires will be included for pharmacoeconomic analysis from an institutional perspective. DISCUSSION/SIGNIFICANCE OF IMPACT: There are no studies investigating the clinical utility of implementing guided anti-platelet selection, combining *CYP2C19* genotyping and HPR phenotyping. We anticipate incorporating this precision medicine approach to guide P2Y_12_ inhibitor selection will be feasible while improving patient outcomes.

